# Retroperitoneal Abscess Revealing Ascending Colon Adenocarcinoma: A Case Report

**DOI:** 10.7759/cureus.88656

**Published:** 2025-07-24

**Authors:** Evangelia Dimitrakopoulou, Paraskevas Stamopoulos, Grigorios Mitsopoulos, Ourania Batsi, Ioannis Kaklamanos

**Affiliations:** 1 University of Athens Department of Surgery, General and Oncological Hospital of Kifissia "Agioi Anargyroi", Athens, GRC; 2 Department of Pathology, General and Oncological Hospital of Kifissia "Agioi Anargyroi", Athens, GRC; 3 Department of Surgery, National and Kapodistrian University of Athens, Athens, GRC

**Keywords:** adenocarcinoma, colorectal cancer, retroperitoneal abscess, right hemicolectomy, tumor perforation

## Abstract

Colon cancer is a common malignancy that can pose significant diagnostic and therapeutic challenges, especially when complicated by atypical presentations such as retroperitoneal abscess formation. We present the case of a 50-year-old Caucasian man with no significant medical history who initially presented in the emergency department due to a right lumbar abscess that had been incised at another facility. Imaging revealed a retroperitoneal abscess confined to the retroperitoneum. Initial conservative management with antibiotics and drainage was undertaken; however, subsequent imaging revealed communication with the ascending colon. The patient subsequently underwent a right hemicolectomy. Histopathological analysis confirmed a moderately differentiated adenocarcinoma of the colon, and the patient was referred for oncological evaluation. This case highlights the importance of considering underlying malignancy in atypical abscess presentations and emphasizes the role of advanced imaging and timely surgical intervention in improving outcomes.

## Introduction

Colorectal cancer is a prevalent malignancy, and due to its high incidence, standardized preventive screening protocols - such as fecal occult blood testing, sigmoidoscopy, and colonoscopy - are now widely implemented, while recent advancements in evidence-based and standardized treatments have notably improved patient survival rates and quality of life [1, 2]. Although often asymptomatic, colon cancer may present with symptoms of fatigue, blood in the stool, abdominal pain, or obstructive ileus [3], the latter indicating an advanced stage. In the general population, colorectal carcinoma should be considered in patients over 40 years old who show atypical signs of appendicitis, along with symptoms such as weight loss, anemia, prolonged illness, and postoperative fecal fistulas, although in younger patients, the probability of colon cancer is lower [4].

Given that the colon is an intraperitoneal organ, with the ascending and descending colon situated behind the peritoneum [5], tumors at these locations may lead to retroperitoneal abscesses due to local invasion and perforation [4, 6-9]. The occurrence of retroperitoneal abscesses resulting from colon cancer is rare [10], with an estimated incidence of 0.3% according to some reports [2], a high mortality rate of approximately 20% [11], and more frequently concerning older patients with comorbidities presenting heterogeneous signs and symptoms [12, 13].

Abscesses stemming from left-sided colon cancer are more likely to remain localized, while those arising from right-sided colon cancer tend to spread to the subcutaneous tissues of the waist and buttocks. Most unusual signs of retroperitoneal abscesses involve subcutaneous emphysema with pneumomediastinum and psoas abscesses [12]. Based on most published cases, the highest point of abscess formation is at the origin of the iliopsoas muscle, with the lowest being from the adductor muscle group to the knee [2].

## Case presentation

**Figure 1:**
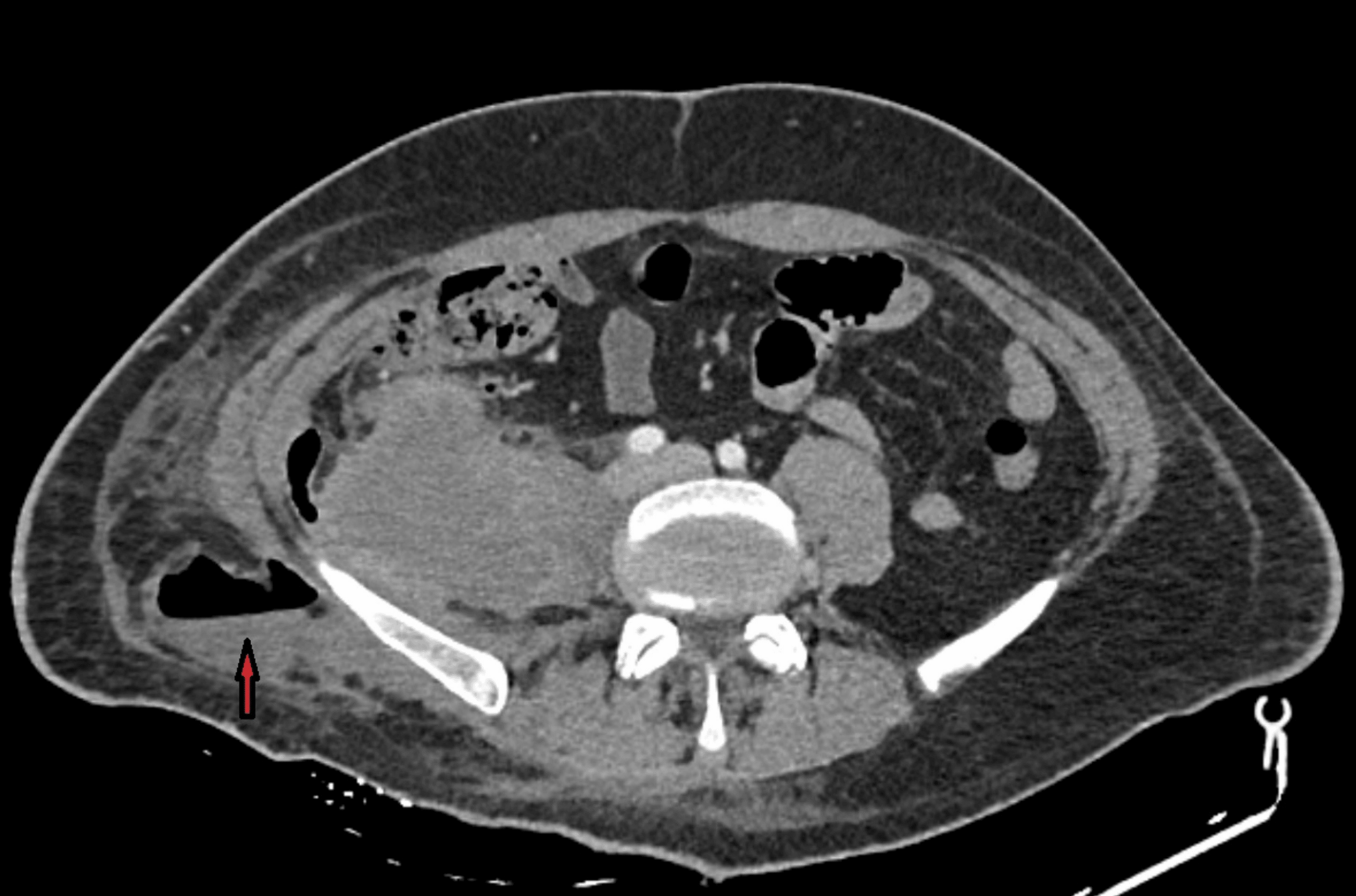
CT findings at presentation revealing the retroperitoneal abscess (red arrow).

We describe a case of a 50-year-old Caucasian man with no significant medical history who presented to the emergency department due to an apparently subcutaneous abscess at the right lumbar region, which had been incised in the emergency department of another hospital 24 hours earlier, and no other symptoms. Τhe patient was hemodynamically stable and afebrile. By the time of our examination, persistent purulent drainage was noted. The patient was admitted to the Surgical Department for antibiotic treatment with clindamycin and amoxicillin and further evaluation. Laboratory results showed an elevated white blood cell count (WBC 13.85 x 103 K/μl) and C-reactive protein (CRP 15.38 mg/dL), and pus culture isolated *Staphylococcus epidermidis, Enterococcus faecalis, and Streptococcus anginosus*. A Computed Tomography (CT) scan of the upper and lower abdomen with intravenous contrast was performed, which revealed an abscess extending from the right retroperitoneal area without contact with the intraperitoneal cavity, through the iliopsoas muscle to the skin of the right lumbar region (Figure 1).

**Figure 2:**
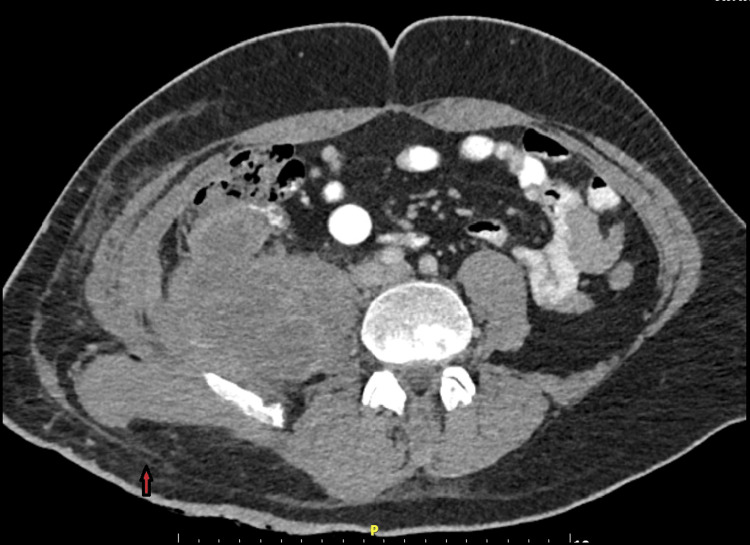
CT findings of follow-up examination revealing the abscess drainage area (red arrow).

On the 7th day of hospitalization, a follow-up CT scan of the upper and lower abdomen was performed, and according to the results, no further intervention was deemed necessary as the report indicated that a significant portion of the abscess had already been drained, and there was an existing natural drainage channel (Figure 2).

**Figure 3:**
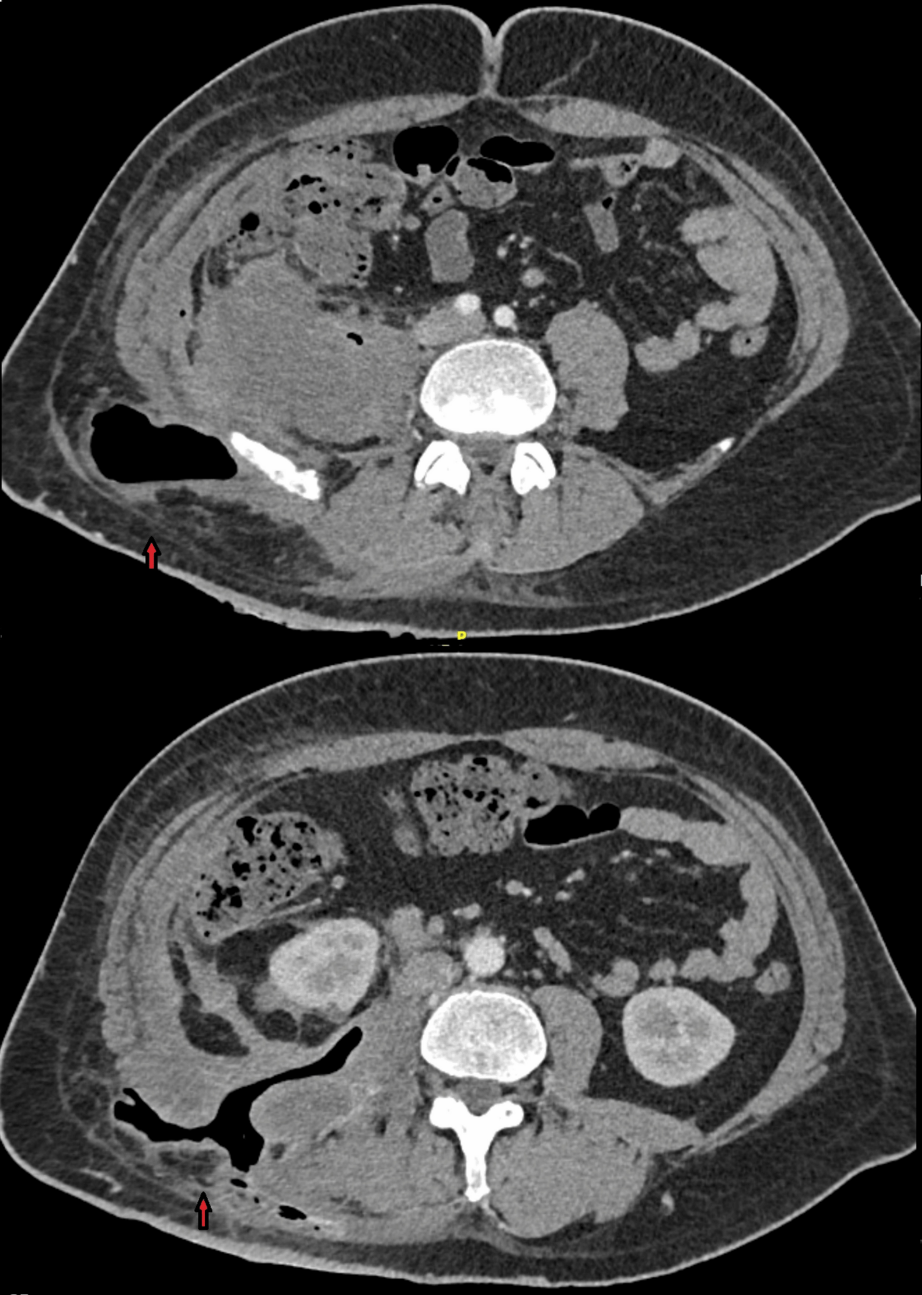
Postoperative CT findings revealing the drain track (red arrow).

**Figure 4:**
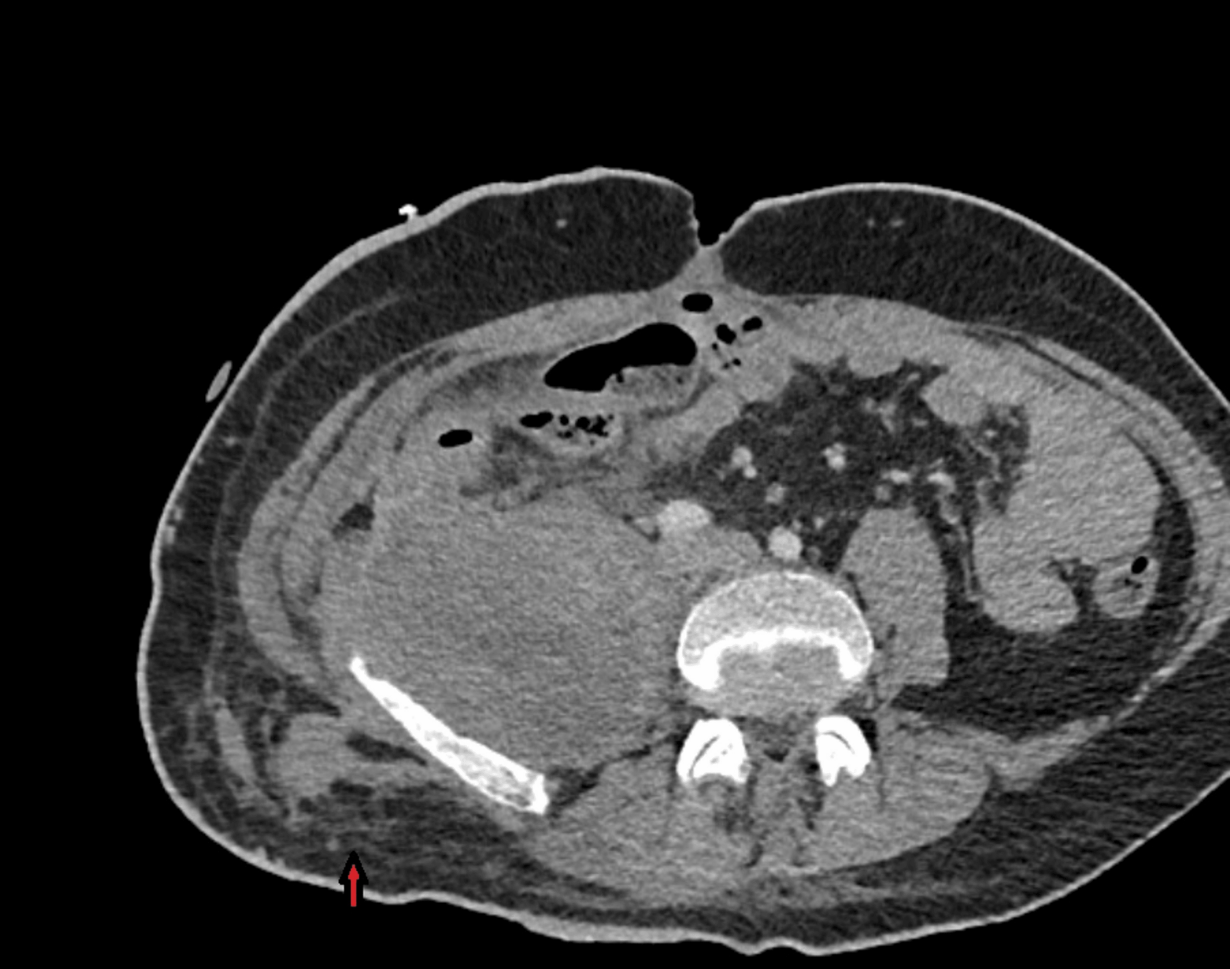
Postoperative CT imaging after 1 year showing the healed area (red arrow).

All inflammatory markers were within normal limits. The patient was discharged with a drainage bag in the right lumbar region, antibiotic coverage, and a recommendation for follow-up imaging in two weeks. The new imaging with CT scan, as well as the non-decreasing drainage of the abscess cavity, indicated possible contact of the abscess with the ascending colon. A Magnetic Resonance (MR) Enterography was performed, which revealed possible contact between the abscess and the ascending colon, without any further complications concerning the intraperitoneal cavity. The patient was immediately taken to the operating room. During the surgery, a large inflammatory mass was found in the cecum and ascending colon, which was firmly adherent to the posterior peritoneum but not to the adjacent organs. Upon mobilizing this mass, the abscess cavity was revealed, and a right hemicolectomy was performed with primary side-to-side ileotransverse anastomosis, and a drainage was placed on the site of the right lumbar region. The patient was discharged on the 7th postoperative day with the drainage. The histological examination revealed moderately differentiated adenocarcinoma of the large intestine, with immunohistochemical analysis: CK7 (+), CK20 (+), CDX-2 (+), GATA-3 (-), P63 (-), NKX3.1 (-), and PSAP (-). All 14 lymph nodes excised were negative for malignancy. The final pathologic classification was T4bN0M0. The patient was further scheduled for an oncologic consultation after our hospital’s oncology board. Figure 3 and Figure 4 show the postoperative image one month and one year after the immunotherapy and chemotherapy, respectively.

## Discussion

Diagnosing colon cancer when it presents as a retroperitoneal abscess can be difficult as a rare cause [12, 14, 15] and is often linked with a poor prognosis [6], especially due to the non-specific signs, resulting in diagnostic and therapeutic delays [10, 16]. The main reasons for large bowel perforation are colorectal cancer and colonic diverticulitis [2, 12, 14, 17, 18]. There are significant differences between abscesses caused by colorectal cancer and those caused by diverticula: retroperitoneal abscesses from colorectal cancer, typically take longer to develop [2], with an incidence that ranges from 3% to 10% [12], while diverticular abscesses are more common, especially when they are left-sided [12, 17] and often appear suddenly and exhibit more widespread retroperitoneal diffusion [2].

Iliopsoas and iliacus abscesses typically result from hematogenous and lymphatic infections or from inflammation spreading from nearby organs [19]. They are commonly seen in patients with chronic illnesses such as diabetes and kidney disease, drug abuse, or immunosuppression [19]. However, our patient had no significant medical history. Although many tumors in the right lower quadrant initially present as peri-appendiceal abscesses [5], carcinomas should also be considered in the differential diagnosis and more specifically ascending colon and ileocecal neoplasms [8, 10]. It has been reported that timely intervention can prevent potential complications such as necrotizing fasciitis [15, 20].

Preoperative imaging, such as CT, MRI, ultrasound, and X-ray, is crucial for assessing colorectal cancer associated with retroperitoneal abscesses [2]. In general, patients with presumed or proven colon cancer should undergo a full colonic evaluation [3]. According to recent studies and case reports, CT scans in patients with retroperitoneal abscesses secondary to colorectal cancer frequently show variably sized collections that compress surrounding tissues and mask underlying intestinal tumors, making diagnosis more difficult [2, 4, 6, 8-10, 12, 18, 19].

In some cases, conventional imaging techniques have clear limitations in evaluating important tumor characteristics [21]. In the past few decades, technological and artificial intelligence revolutions have evolved imaging procedures; thus, it can be regarded as crucial to integrate these techniques into our diagnostic algorithm, especially for more complicated cases. CT colonography and colon capsule endoscopy are alternative techniques, with diagnostic performance superior to barium enema [21]. It is shown that these two techniques have been able to detect meaningful colonic mucosal lesions in about 11% to 13% with CT colonography and up to 24% to 44% with colon capsule endoscopy of patients who had previous incomplete colonoscopy [3]. They can be considered strongly in cases where we cannot use conventional colonoscopy; for example, when the general assessment suggests a possible colonic perforation.

For patients with suspected right-sided cancer, laparoscopic right colectomy is often preferred due to its minimally invasive nature and potential curative outcome [3, 22]. In cases of tumor invasion, however, additional surgical approaches may be needed. Complications such as perforation, bleeding, obstruction, and abscess formation may arise [2, 4, 6-10, 12, 15, 18, 19]. Moreover, if the tumor invades nearby organs, there is a risk of cancer cell spillage during surgical separation, which is linked to poorer outcomes [22]. Managing these complications and transitioning promptly to cancer treatment are critical for improving prognosis [2, 18].

In terms of abscess management, conservative approaches, such as CT-guided drainage, are generally preferred for intraperitoneal abscesses. However, when colorectal cancer is the underlying cause, treatment becomes more complex. While incision, drainage, and antimicrobial therapy can relieve abscess symptoms, a surgical approach for the tumor is a definite solution and prevention for further complications [2, 18]. Direct colon resection, combined with intraoperative debridement and drainage, may increase the risk of intraperitoneal abscess spread [2]. Ideally, a comprehensive treatment plan addressing both the abscess and the cancer, followed by further oncological treatment, provides the most effective long-term prognosis [2].

## Conclusions

Retroperitoneal abscesses secondary to colorectal cancer are exceptionally rare and pose significant diagnostic and therapeutic challenges. This case underscores the importance of maintaining a high index of suspicion, particularly when an abscess is unusual in location or drainage fails to resolve with appropriate management. Timely and accurate diagnosis requires a multidisciplinary approach, integrating advanced imaging modalities to identify occult colonic malignancies. While initial management may involve conservative drainage, definitive treatment hinges on addressing the underlying neoplasm through surgical resection. Early recognition and coordinated care are essential to reduce complications, ensure oncologic control, and improve overall patient outcomes.
